# Postmarketing Follow-Up of a Digital Home Exercise Program for Back, Hip, and Knee Pain: Retrospective Observational Study With a Time-Series and Matched-Pair Analysis

**DOI:** 10.2196/43775

**Published:** 2023-02-27

**Authors:** Gisbert Wilhelm Teepe, Tobias Kowatsch, Felix Patricius Hans, Leo Benning

**Affiliations:** 1 Centre for Digital Health Intervention ETH Zürich Zürich Switzerland; 2 Institute for Implementation Science in Health Care University of Zürich Zürich Switzerland; 3 School of Medicine University of St. Gallen St.Gallen Switzerland; 4 University Emergency Center Medical Center - University of Freiburg Freiburg Germany; 5 Faculty of Medicine University of Freiburg Freiburg Germany; 6 Vivira Health Lab GmbH Berlin Germany

**Keywords:** digital therapeutics, digital health, musculoskeletal diseases, exercise training

## Abstract

**Background:**

Musculoskeletal conditions are the main drivers of global disease burden and cause significant direct and indirect health care costs. Digital health applications improve the availability of and access to adequate care. The German health care system established a pathway for the approval of “Digitale Gesundheitsanwendungen” (DiGAs; Digital Health Applications) as collectively funded medical services through the “Digitale-Versorgung-Gesetz” (Digital Health Care Act) in 2019.

**Objective:**

This article presents real-world prescription data collected through the smartphone-based home exercise program “Vivira,” a fully approved DiGA, regarding its effect on self-reported pain intensity and physical inability in patients with unspecific and degenerative pain in the back, hip, and knee.

**Methods:**

This study included 3629 patients (71.8% [2607/3629] female; mean age 47 years, SD 14.2 years). The primary outcome was the self-reported pain score, which was assessed with a verbal numerical rating scale. The secondary outcomes were self-reported function scores. To analyze the primary outcome, we used a 2-sided Skillings-Mack test. For function scores, a time analysis was not feasible; therefore, we calculated matched pairs using the Wilcoxon signed-rank test.

**Results:**

Our results showed significant reductions in self-reported pain intensity after 2, 4, 8, and 12 weeks in the Skillings-Mack test (T_3628_=5308; *P*<.001). The changes were within the range of a clinically relevant improvement. Function scores showed a generally positive yet more variable response across the pain areas (back, hip, and knee).

**Conclusions:**

This study presents postmarketing observational data from one of the first DiGAs for unspecific and degenerative musculoskeletal pain. We noted significant improvements in self-reported pain intensity throughout the observation period of 12 weeks, which reached clinical relevance. Additionally, we identified a complex response pattern of the function scores assessed. Lastly, we highlighted the challenges of relevant attrition at follow-up and the potential opportunities for evaluating digital health applications. Although our findings do not have confirmatory power, they illustrate the potential benefits of digital health applications to improve the availability of and access to medical care.

**Trial Registration:**

German Clinical Trials Register DRKS00024051; https://drks.de/search/en/trial/DRKS00024051

## Introduction

Globally, musculoskeletal conditions are among the 10 most important drivers of an increasing disease burden and are common in all age groups [1]. Among these conditions, nonspecific lower back pain accounted for 2.5% of all disability-adjusted life years globally in 2019, an increase of 46.9% compared to the 1990 baseline [1]. In the German health care system, musculoskeletal conditions are among the most frequent chronic conditions [2] and constitute a major cause of chronic pain, physical disability, and decreased quality of life [3]. Consequently, musculoskeletal conditions account for significant direct health care expenses and cause relevant indirect health care expenses. Related work estimates that the cost of lost productivity in the European Union due to musculoskeletal conditions is as high as 2% of the European Union’s gross domestic product [4].

For degenerative and nonspecific musculoskeletal conditions, movement therapy and patient education are considered first-line treatment components of international guidelines for managing musculoskeletal conditions [5,6]. However, access to and availability of movement therapy are limited [7]. Digital home exercise applications intend to substitute or support conventional in-person movement therapy to address this challenge. In this context, we present the first postmarketing follow-up data of the standalone digital therapeutic app “Vivira,” a fully approved “Digitale Gesundheitsanwendung” (DiGA; Digital Health Application) under the “Digitale-Versorgung-Gesetz” (DVG; Digital Health Care Act) in Germany [8,9]. While a comprehensive introduction to the German health care system can be found elsewhere [10], several key elements of the health care system and the DVG legislation should be briefly introduced here. Membership in one of the statutory health insurances is mandatory for all individuals with employment in Germany up to an annually adjusted income threshold. Above this threshold, insured individuals can opt out for private insurance. Similar rules exist for self-employed and some specified groups (eg, federal or state employees), while an extended solidarity-funded coverage applies to family members of regularly insured (eg, children or disabled family members), retired, and unemployed individuals. Premiums are generally defined risk-independent and are based on the insured individual’s gross income. While every individual is free to choose among the different statutory health insurances and no risk-based selection by the insurances is allowed, all statutory health insurances have the legal obligation to cover the same collectively contracted benefits package. This comprehensive insurance system covers approximately 90% of the population in Germany. The DVG from 2019 constituted a significant innovation for the German health care system, as it introduced digital therapeutics into German social law. It included the category of DiGA into the collectively contracted benefits package of the statutory health insurances [11]. Hence, all statutory health insurances have the obligation to reimburse these digital therapeutics when prescribed by a qualified health care professional. To receive market approval as a DiGA, however, the digital therapeutics need to meet quality and safety criteria and need to demonstrate relevant medical effectiveness as outlined by the Bundesinstitut für Arzneimittel und Medizinprodukte (BfArM), a regulatory body for drugs and medical devices in Germany [12]. One important feature of the assessment process is the so-called “fast track” approval, which allows preliminary approval for distribution and reimbursement of the respective DiGA for up to 12 months once the quality and safety criteria are met and the successful scientific evaluation is not yet completed, but deemed likely by the authorities. Over the duration of the preliminary approval period, the manufacturer of the DiGA must provide sufficient evidence for the medical effectiveness of the proposed DiGA. If the demonstration fails, the DiGA is not granted permanent listing and the preliminary market approval is withdrawn.

Although the regulatory requirements welcome innovative and real-world evidence-based approaches toward evidence generation, all successful attempts at receiving permanent market approval have so far relied on conventional randomized controlled trials [13,14]. Nonetheless, there is a growing interest in real-world observation data from permanently listed DiGAs to better understand prescription, use, and outcome data under nontrial conditions. This study hence aimed to assess the effects of the DiGA Vivira on self-reported pain intensity and function scores in a real-world setting.

## Methods

### Recruitment

We performed a retrospective observational study based on self-reported pain scores, function scores, and retention data. Besides these outcomes, patients also reported demographic information (age and sex), pain area, and pain duration at baseline. We used data reported by the patients between October 20, 2020, and June 22, 2021, and included all available software versions of Vivira. All patients consented to the use of their data in this study under article 4 of the “Digitale-Gesundheitsanwendungen-Verordnung” (DiGAV; Digital Health Applications Act). All collected data were stored according to the German Data Protection Regulations (Datenschutz-Grundverordnung). We registered the study with the German Center for Clinical Trials (Deutsches Register Klinischer Studien, reference DRKS00024051). Enrollment for the treatment with Vivira was solely at the respective physician’s discretion and without any control from the manufacturer. The inclusion criteria are presented in Textbox 1.

According to the inclusion criteria, we included 3629 patients who could be analyzed with at least one completed assessment after enrollment.

**Inclusion criteria.**

1. Enrollment after preliminary approval of the home exercise program Vivira as a “Digitale Gesundheitsanwendungen” (DiGA; Digital Health Application).
2. A reported initial pain score on a verbal numerical rating scale (range 0-10) of >0.
3. Any reported pain duration (acute, subacute, or chronic).
4. Completion of >0 exercises during participation.
5. Presence of at least two patient-reported data entries.

### Ethics Approval

The study and the underlying evaluation concept received approval from the Ethics Committee of the Medical Association of the state of Baden-Württemberg (Ethikkommission der Landesärztekammer Baden-Württemberg, F-2021-010).

### Physical Exercise Composition and Progression Modules

Upon enrollment, the app prompts participants to complete an initial assessment, which assesses the current functional state (ie, limitations in strength, mobility, and coordination) through a series of exercises that participants can either complete or fail to complete (ie, a binary assessment through different movement exercises). Specific extensions to the assessment account for participants’ pre-existing movement limitations (eg, inability to complete assessment prompts requiring 90° flexion of the hip and knee joints, inability to maintain a stable resting position, or inability to sit on the heels). The completion and noncompletion of each test are assigned weights that allow the computation of function scores for strength, mobility, and coordination. The definition of each weight is based on an interdisciplinary expert panel of orthopedic surgeons and physiotherapists. The underlying principle of the initial assessment follows the therapeutic concept of regional interdependence, which has been described in detail elsewhere [15,16]. In brief, it formalizes the clinical observation that in the context of musculoskeletal conditions, therapeutic interventions applied to one anatomical region can have positive effects on pain and range of motion in other anatomical regions.

**Figure 1:**
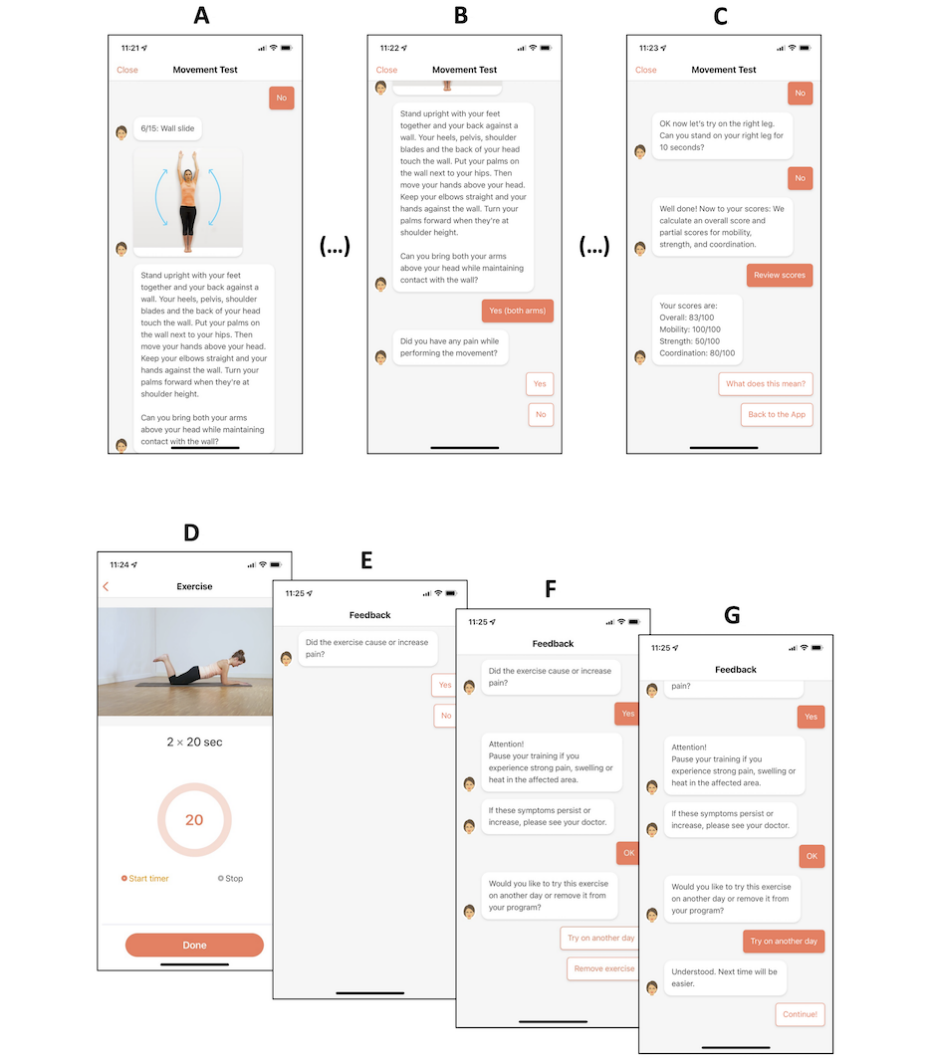
User interface of Vivira. (A) The home screen prompts patients to enter their daily exercise program. (B) Four exercises, composed on the basis of close patient-feedback loops, are displayed and can be entered in any order. (C) Prior to the start of each exercise, video- and text-based instructions explain each exercise in detail, highlight important components of each exercise, and provide background information. (D) During each exercise, a video loop repeats the exercise instructions and displays the number of repetitions or, if applicable, a timer. (E-G) After completion of all 4 daily exercises, the program collects patient feedback (not shown here) and returns to the home screen.

Once a participant completes the initial assessment, the app automatically composes an individualized set of 4 exercises from a repository of 120 different exercises. Every exercise includes a 2-dimensional progression module. The exercise intensity is increased gradually (ie, increases in the number of repetitions and the duration of exercises) before the complexity of the exercise is increased (ie, adding a sequence of exercise changes or adding another movement component). The app modifies the intensity, complexity, and composition of the exercise according to participant feedback. Participants are required to provide binary feedback after each exercise to ensure that a prompted exercise neither triggers new pain sensations, nor exacerbates existing pain before being prompted with the next exercise. This feedback guides the automated customization process of the exercise program. Figure 1 illustrates the user interface of the app.

### Self-Reported Outcome Measures

The app collects the current and self-assessed pain intensity based on a verbal numerical rating scale (VNRS) [17] once per week.

Function scores are based on the initial assessment of the functional state, as outlined above. A reassessment is prompted to participants every 4 weeks as a virtual follow-up. Upon completion, an updated functional state along the 3 dimensions of strength, mobility, and coordination is provided. Additionally, a composite (total) score is computed.

### Statistical Methods

The hypothesis test used for self-reported pain intensity was the 2-sided Skillings-Mack test, which is particularly useful for an unbalanced and incomplete block design or in the presence of missing data due to design or missing at random. For function scores, a meaningful time analysis was not feasible due to high attrition for completed movement assessments and, therefore, we calculated matched pairs. We used the Wilcoxon signed-rank test as the hypothesis test and calculated the IQR. We used median intervals (days) between completed functional reports to form cohorts, which we referred to as “first-to-second entry,” “first-to-third entry,” and “first-to-fourth entry.” All participants were matched to themselves at baseline at the respective time of each entry. We used the Bonferroni method to control for family-wise errors and report corrected alpha levels for the Skillings-Mack test and Wilcoxon signed-rank test.

We performed chi-square tests to investigate the differences at baseline in pain area (ie, upper back, lower back, hip, or knee) and pain duration (ie, acute, subacute, or chronic) among participant age groups. TTo illustrate standardized residuals for each chi-square test, we presented mosaic plots. We recorded overall pain scores and classified pain duration at the time of enrollment according to global consensus [18,19].

## Results

### User Statistics

A total of 3629 patients met the inclusion criteria and provided at least two data points needed for comparison with an intraindividual control over 12 weeks. We formed age groups to investigate differences between age groups in pain duration and pain area. Table 1 presents the baseline characteristics of the patients.

**Figure 2:**
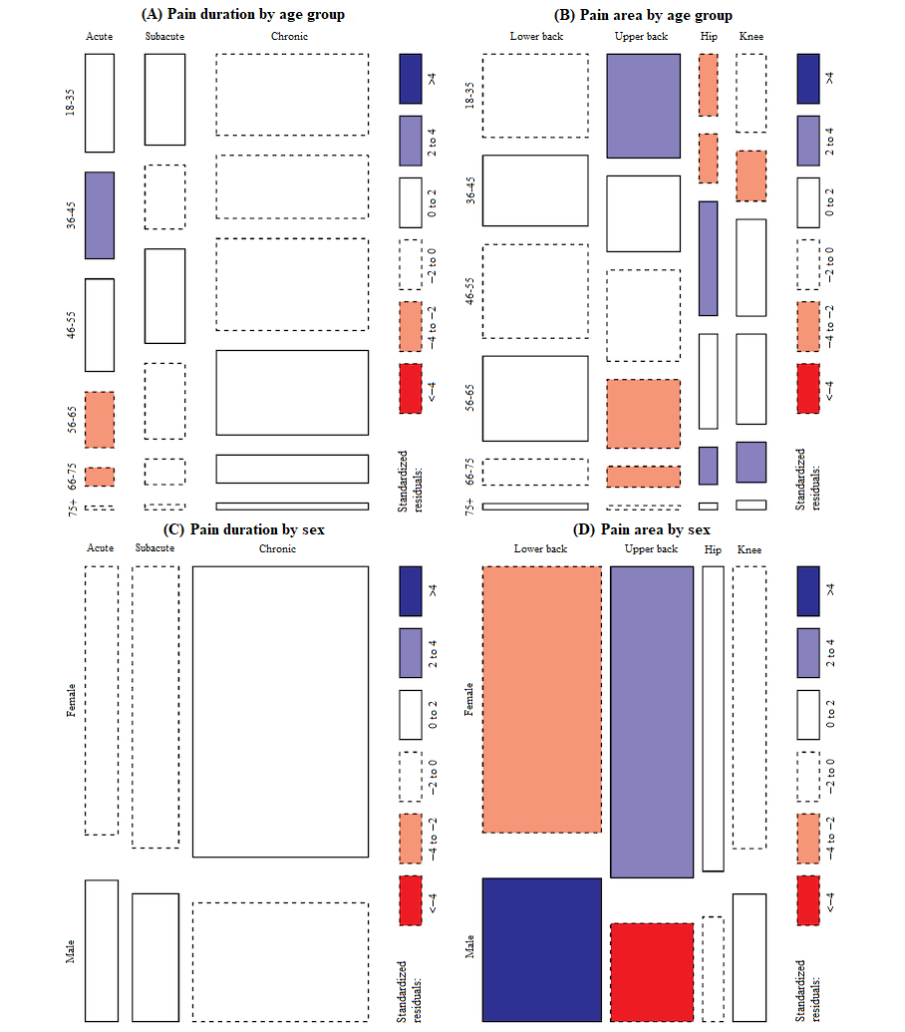
Mosaic plot illustrating the baseline distribution for age (A and B) or sex (C and D) by pain duration or pain area. The size of the square illustrates the number of observations. A larger square indicates the size of the observations regarding age or sex with the corresponding pain duration or pain area. The color indicates which direction this specific observation differs from the expected observation denoted by the standardized residuals. The color depth indicates how strongly the specific observation differs from the expected observation denoted by the standardized residuals.

Using chi-square tests, the number of female or male patients did not significantly differ over 4 assessments (*χ*^2^_4_=1.9; *P*=.75). Additionally, we investigated whether the 2 main demographic features of age group and sex influenced pain duration and pain area. We observed a significant association between pain duration and age group (*χ*^2^_10_=24.36; *P*<.001). Moreover, we observed a significant association between sex and both pain duration (*χ*^2^_2_=12.09; *P*=.002) and pain area (*χ*^2^_3_=33.73; *P*<.001). Figure 2 illustrates these findings, and Multimedia Appendix 1, Multimedia Appendix 2, Multimedia Appendix 3, and Multimedia Appendix 4 further describe these results using contingency tables.

**Table 1:** Baseline characteristics of the study population.

Age group and sex		Total patients, n	Pain duration, n				Reported pain area, n			
			Acute	Subacute	Chronic	Lower back		Upper back	Hip	Knee
**18-35 years**		891	124	180	587	397		343	46	105
	Female	597	75	115	407	259		248	29	61
	Male	294	49	65	180	138		95	17	44
**36-45 years**		672	102	122	448	313		257	36	66
	Female	474	66	80	328	203		201	29	41
	Male	198	36	42	120	110		56	7	25
**46-55 years**		954	106	187	661	425		302	97	130
	Female	733	80	148	505	306		249	80	98
	Male	221	26	39	156	119		53	17	32
**56-65 years**		813	73	147	593	386		229	75	123
	Female	591	45	106	440	263		173	59	96
	Male	222	28	41	153	123		56	16	27
**66-75 years**		248	21	49	178	101		65	28	54
	Female	183	14	37	132	70		52	21	40
	Male	65	7	12	46	31		13	7	14
**>75 years**		51	4	9	38	20		13	6	12
	Female	29	1	4	24	12		8	3	6
	Male	22	3	5	14	8		5	3	6

### Assessment of Patient-Reported Pain Intensity

**Figure 3:**
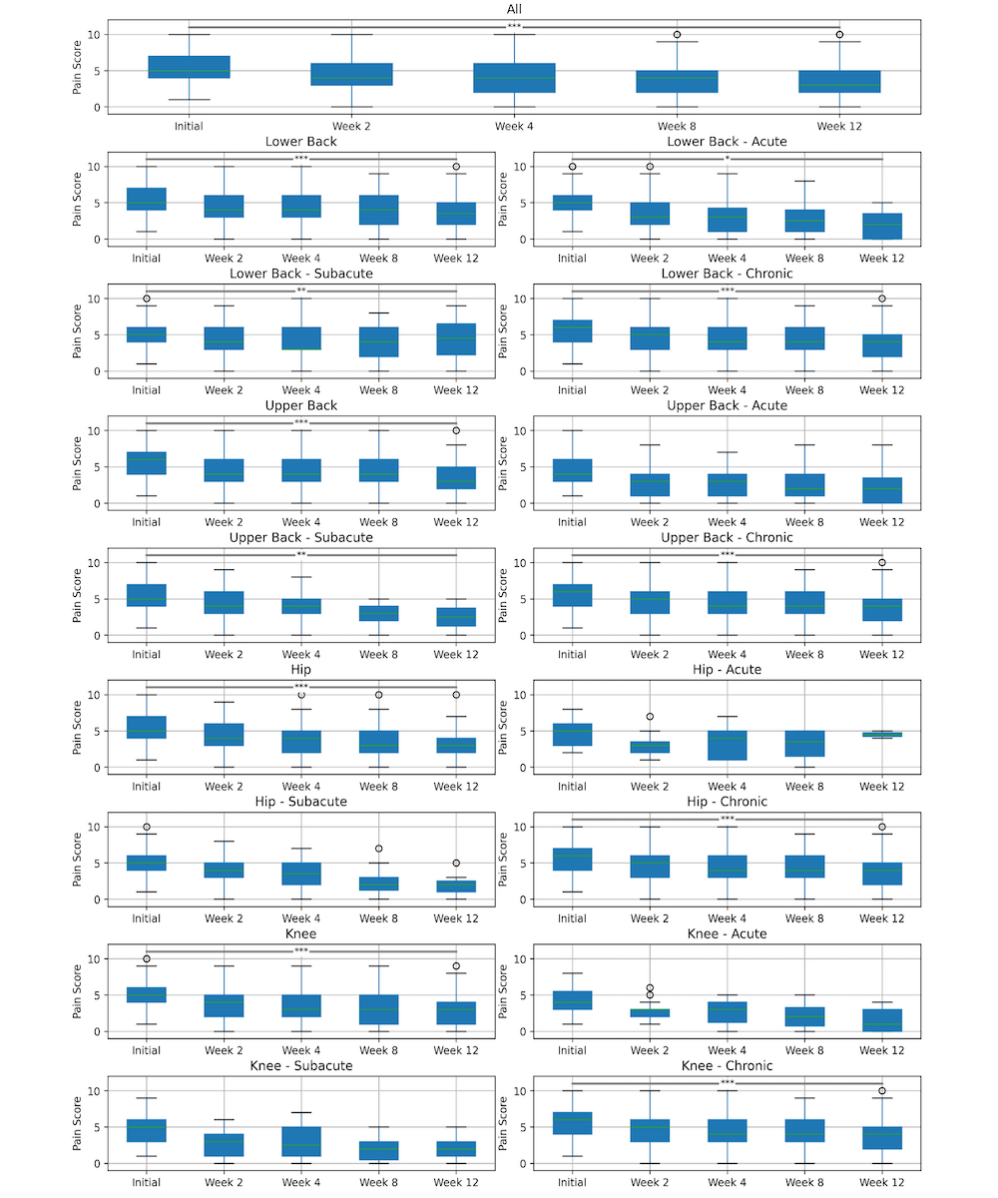
Average self-reported pain score for each retention time period for all pain areas, specific pain areas, and pain areas and durations. The center line (green) indicates the median, boxplot limits indicate the upper and lower quartiles, whiskers indicate the 1.5× interquartile range, and points indicate outliers. *P<.05, **P<.005, ***P<.0005 (Skillings-Mack test).

Prior to the formation of indication-specific strata, we noted a substantial reduction in pain scores across 2, 4, 8, and 12 weeks (T_3628_=5308; *P*<.001). The mean pain intensity values (out of 10) at baseline, 2 weeks, 4 weeks, 8 weeks, and 12 weeks were 5.42 (SD 1.79), 4.36 (SD 2.21), 3.99 (SD 2.22), 3.84 (SD 2.27), and 3.48 (SD 2.36), respectively. Figure 3 and Table 2 illustrate these differences and report additional stratum-specific (ie, for different pain areas and pain durations) results.

**Table 2:** Self-reported pain scores and changes across indication subsets and reported pain durations by retained days.

Pain area			Initial					Week 2					Week 4					Week 8					Week 12					Skillings-Mack test			
			n		Mean (SD)		n			Mean (SD)		n			Mean (SD)		n			Mean (SD)		n			Mean (SD)		Test values (*df*)			Pain reduction, %	
All			3629		5.42 (1.79)		1776			4.36 (2.21)		1330			3.99 (2.22)		820			3.84 (2.27)		458			3.48 (2.36)		5308.10 (3628)^a^			−35.86	
**Lower back**			1642		5.43 (1.76)		812			4.44 (2.20)		601			4.08 (2.22)		376			4.02 (2.23)		207			3.78 (2.45)		2369.86 (1641)^a^			−30.39	
	Acute	231		4.87 (1.86)		117			3.62 (2.28)		66			2.85 (2.35)		42			2.57 (2.17)		19			2.09 (1.97)		304.46 (230)^b^			−57.10		
	Subacute	314		5.21 (1.64)		149			4.30 (2.15)		112			3.98 (2.34)		64			4.03 (2.22)		31			4.39 (2.52)		423.39 (313)^c^			−15.76		
	Chronic	1097		5.62 (1.74)		546			4.66 (2.16)		423			4.29 (2.11)		270			4.22 (2.18)		157			3.86 (2.43)		1620.33 (1096)^a^			−31.20		
**Upper back**			1209		5.59 (1.78)		573			4.51 (2.23)		419			4.20 (2.26)		249			4.09 (2.27)		129			3.58 (2.34)		1717.08 (1208)^a^			−35.98	
	Acute	140		4.50 (1.67)		61			2.88 (2.03)		52			2.63 (2.00)		28			2.71 (2.39)		18			2.55 (3.01)		171.14 (139)^d^			−43.43		
	Subacute	207		5.40 (1.69)		94			4.23 (2.29)		75			3.84 (1.83)		35			3.00 (1.44)		22			2.60 (1.71)		285.51 (206)^c^			−51.86		
	Chronic	862		5.82 (1.75)		418			4.82 (2.13)		292			4.56 (2.29)		186			4.50 (2.24)		89			3.98 (2.21)		1216.06 (861)^a^			−31.57		
**Hip**			288		5.36 (1.75)		159			4.35 (2.20)		122			3.80 (1.98)		73			3.42 (2.16)		45			3.00 (2.16)		441.23 (287)^a^			−44.04	
	Acute	24		4.63 (1.95)		11			3.18 (1.66)		14			3.54 (2.15)		7			3.00 (2.45)		4			4.50 (0.71)		34.01 (23)^d^			−2.70		
	Subacute	66		5.06 (1.61)		38			3.97 (2.20)		26			3.35 (1.96)		12			2.50 (2.17)		10			2.00 (1.63)		97.65 (65)^d^			−60.48		
	Chronic	198		5.55 (1.74)		110			4.60 (2.20)		82			4.00 (1.96)		54			3.69 (2.12)		31			3.21 (2.32)		298.20 (197)^a^			−42.16		
**Knee**			490		4.97 (1.85)		232			3.71 (2.09)		188			3.41 (2.20)		122			3.20 (2.31)		77			2.87 (2.15)		744.03 (489)^a^			−42.19	
	Acute	35		4.46 (1.72)		15			2.73 (1.44)		14			2.79 (1.72)		9			2.13 (1.81)		7			1.60 (1.82)		45.47 (34)^d^			−64.10		
	Subacute	107		4.63 (1.73)		52			2.85 (1.69)		36			2.88 (2.21)		23			1.89 (1.56)		13			2.11 (1.45)		138.91 (106)^d^			−54.37		
	Chronic	348		5.12 (1.88)		165			4.07 (2.16)		138			3.61 (2.22)		90			3.63 (2.37)		57			3.20 (2.26)		544.55 (347)^a^			−37.51		

^a^Adjusted *P*<.0005 (calculated using Bonferroni correction).

^b^Adjusted *P*<.05 (calculated using Bonferroni correction).

^c^Adjusted *P*<.005 (calculated using Bonferroni correction).

^d^Adjusted *P*>.05 (calculated using Bonferroni correction).

### Patient-Reported Functional Assessment

Chronic conditions appeared to improve along the dimensions of strength, mobility, and coordination, as well as the total score (Tables 3-6). This finding was consistent over all intervals of submitted function scores assessed (Multimedia Appendix 5 and Multimedia Appendix 6). Within chronic conditions, only patients with chronic hip pain did not achieve significant improvements in mobility and coordination across any completed submission of function scores (Multimedia Appendix 5 and Multimedia Appendix 6). Overall, the strength score showed significant improvements across most pain areas studied. However, patients with acute lower back pain, acute upper back pain, and acute hip pain did not show significant improvements in strength scores between their first and second assessments of function scores (Tables 3-6).

**Table 3:** Self-reported total function scores and changes across indication subsets and reported pain durations by retained days.

Reported pain area and duration		Retained days, value (IQR)	Initial, value (IQR)	Last, value (IQR)	*P* value^a^
**Lower back**					
	Acute (n=50)	29 (14-32)	67 (50-77)	73 (60-80)	.0028^b^
	Subacute (n=74)	29 (17-33)	63 (47-73)	67 (53-80)	<.0001^c^
	Chronic (n=326)	29 (23-32)	57 (40-73)	67 (50-80)	<.0001^c^
**Upper back**					
	Acute (n=29)	30 (10-33)	67 (53-73)	77 (60-87)	.0001^c^
	Subacute (n=51)	29 (28-36)	60 (37-77)	63 (43-80)	.0120^b^
	Chronic (n=226)	29 (17-33)	53 (33-67)	57 (43-73)	<.0001^c^
**Hip**					
	Acute (n=12)	28 (25.5-29.5)	65 (48.5-70)	71.5 (57-88)	.0566^d^
	Subacute (n=22)	27.5 (13-30)	55 (43-67)	67 (50-80)	.0021^b^
	Chronic (n=70)	29 (28-32)	63 (50-73)	67 (53-80)	.0025^b^
**Knee**					
	Acute (n=12)	29 (28-36)	53 (45-65)	71.5 (53.5-80)	.0371^d^
	Subacute (n=32)	28.5 (23-32.5)	63 (55-73)	77 (63-87)	<.0001^c^
	Chronic (n=107)	30 (27-35)	60 (47-70)	67 (53-80)	<.0001^c^

^a^Adjusted for family-wise error using the Bonferroni method.

^b^*P*<.0167.

^c^*P*<.000167.

^d^Not significant.

**Table 4:** Self-reported strength scores and changes across indication subsets and reported pain durations by retained days.

Reported pain area and duration		Retained days, value (IQR)	Initial, value (IQR)	Last, value (IQR)	*P* value^a^
**Lower back**					
	Acute (n=50)	29 (14-32)	60 (40-80)	70 (50-80)	.0215^b^
	Subacute (n=74)	29 (17-33)	60 (30-70)	60 (40-90)	.0010^c^
	Chronic (n=326)	29 (23-32)	50 (30-70)	60 (40-80)	<.0001^d^
**Upper back**					
	Acute (n=29)	30 (10-33)	60 (50-80)	60 (60-100)	.0198^b^
	Subacute (n=51)	29 (28-36)	50 (20-80)	60 (40-90)	.0076^e^
	Chronic (n=226)	29 (17-33)	50 (20-80)	60 (40-80)	<.0001^d^
**Hip**					
	Acute (n=12)	28 (25.5-29.5)	45 (40-70)	75 (45-85)	.1270^b^
	Subacute (n=22)	27.5 (13-30)	55 (20-60)	65 (40-100)	.0001^d^
	Chronic (n=70)	29 (28-32)	60 (40-80)	70 (50-100)	.0093^e^
**Knee**					
	Acute (n=12)	29 (28-36)	45 (20-70)	60 (45-100)	.0156^e^
	Subacute (n=32)	28.5 (23-32.5)	80 (60-80)	90 (60-100)	.0066^e^
	Chronic (n=107)	30 (27-35)	60 (40-80)	70 (50-100)	.0006^c^

^a^Adjusted for family-wise error using the Bonferroni method.

^b^Not significant.

^c^*P*<.00167.

^d^*P*<.000167.

^e^*P*<.0167.

**Table 5:** Self-reported mobility scores and changes across indication subsets and reported pain durations by retained days.

Reported pain area and duration		Retained days, value (IQR)	Initial, value (IQR)	Last, value (IQR)	*P* value^a^
**Lower back**					
	Acute (n=50)	29 (14-32)	70 (55-80)	75 (65-80)	.0297^b^
	Subacute (n=74)	29 (17-33)	67,5 (45-80)	70 (55-80)	.0006^c^
	Chronic (n=326)	29 (23-32)	60 (45-75)	70 (50-80)	<.0001^d^
**Upper back**					
	Acute (n=29)	30 (10-33)	65 (55-75)	80 (60-90)	.0001^d^
	Subacute (n=51)	29 (28-36)	60 (40-75)	55 (45-80)	.1191^b^
	Chronic (n=226)	29 (17-33)	50 (35-70)	55 (40-75)	<.0001^d^
**Hip**					
	Acute (n=12)	28 (25.5-29.5)	67.5 (52.5-85)	67.5 (60-87.5)	.2578^b^
	Subacute (n=22)	27.5 (13-30)	57.5 (50-70)	62.5 (50-80)	.0251^b^
	Chronic (n=70)	29 (28-32)	60 (50-70)	65 (50-75)	.0201^b^
**Knee**					
	Acute (n=12)	29 (28-36)	60 (52.5-70)	70 (52.5-82.5)	.1426^b^
	Subacute (n=32)	28.5 (23-32.5)	60 (50-70)	72.5 (60-82.5)	<.0001^d^
	Chronic (n=107)	30 (27-35)	60 (45-70)	65 (50-80)	<.0001^d^

^a^Adjusted for family-wise error using the Bonferroni method.

^b^Not significant.

^c^*P*<.00167.

^d^*P*<.000167.

**Table 6:** Self-reported coordination scores and changes across indication subsets and reported pain durations by retained days.

Reported pain area and duration		Retained days, value (IQR)	Initial, value (IQR)	Last, value (IQR)	*P* value^a^
**Lower back**					
	Acute (n=50)	29 (14-32)	70 (60-80)	80 (60-80)	.1368^b^
	Subacute (n=74)	29 (17-33)	70 (50-80)	80 (50-80)	.0766^b^
	Chronic (n=326)	29 (23-32)	65 (40-80)	80 (50-90)	<.0001^c^
**Upper back**					
	Acute (n=29)	30 (10-33)	80 (60-80)	80 (80-100)	.2664^b^
	Subacute (n=51)	29 (28-36)	60 (40-80)	70 (40-80)	.2129^b^
	Chronic (n=226)	29 (17-33)	60 (40-80)	60 (40-80)	.0005^d^
**Hip**					
	Acute (n=12)	28 (25.5-29.5)	60 (55-80)	70 (55-95)	.5000^b^
	Subacute (n=22)	27.5 (13-30)	60 (30-80)	65 (40-80)	.1396^b^
	Chronic (n=70)	29 (28-32)	60 (40-80)	60 (50-80)	.2875^b^
**Knee**					
	Acute (n=12)	29 (28-36)	55 (35-60)	60 (55-60)	.1562^b^
	Subacute (n=32)	28.5 (23-32.5)	60 (50-80)	80 (60-85)	.0066^e^
	Chronic (n=107)	30 (27-35)	60 (40-80)	60 (40-80)	.0026^e^

^a^Adjusted for family-wise error using the Bonferroni method.

^b^Not significant.

^c^*P*<.000167.

^d^*P*<.00167.

^e^*P*<.0167.

The continuation of exercise and the consequent submission of further function scores led to significant improvements in the strength score for only patients with acute lower back pain (Multimedia Appendix 5). Similarly, mobility and coordination scores improved particularly well in patients with chronic conditions, but failed to improve significantly in patients with chronic hip pain. Yet, compared with the pain scores, we did not see a leveling off of the improvements after the first reported interval and saw continuous improvements, particularly in the mobility and strength scores (Multimedia Appendix 5 and Multimedia Appendix 6).

### Assessment of Retention

**Figure 4:**
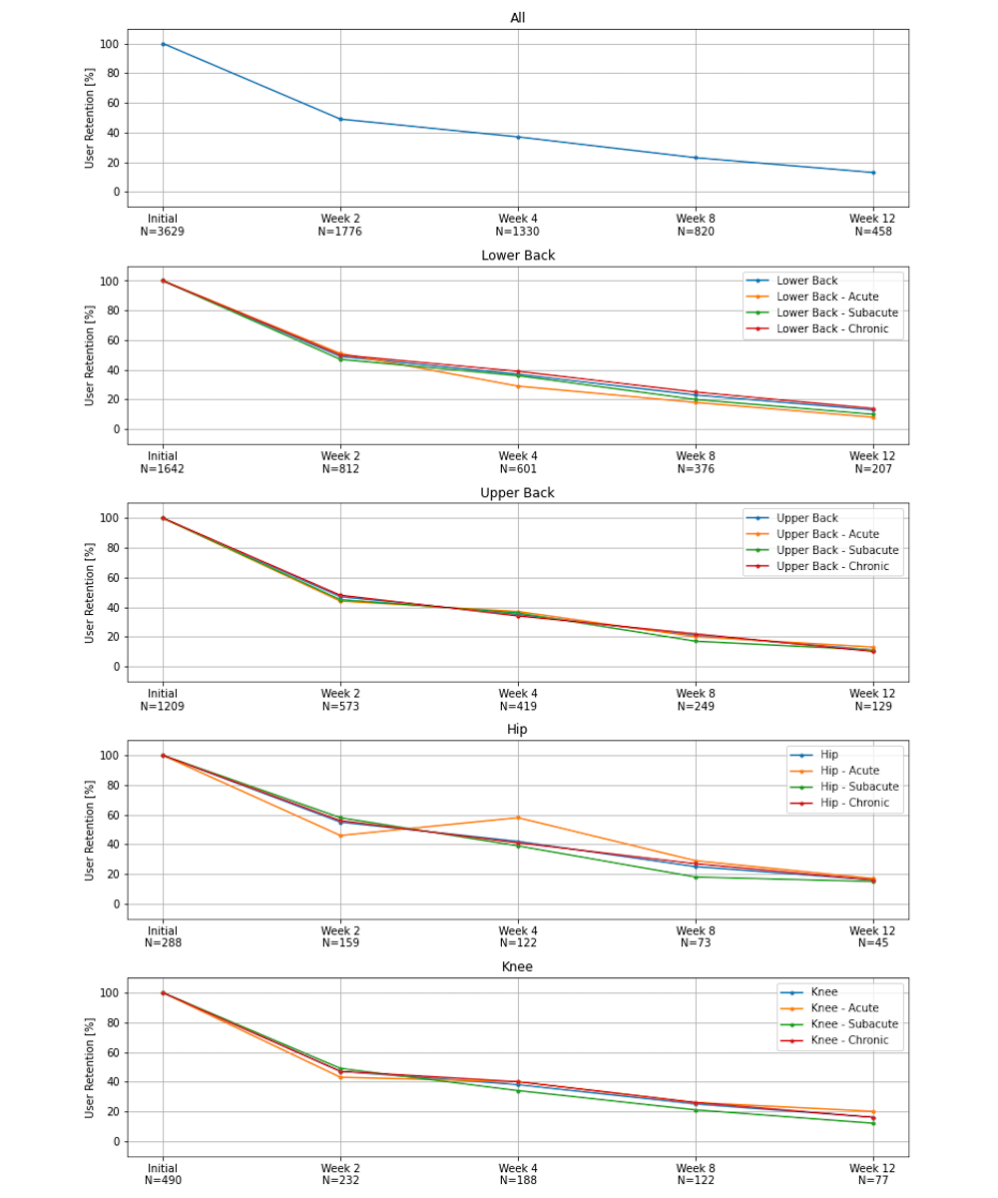
Retention rates for different pain areas and durations. A more detailed overview is provided in Multimedia Appendix 7.

Our analysis showed that the home exercise app Vivira achieved overall retention rates of 36.6% (601/1642) for lower back pain, 34.7% (419/1209) for upper back pain, 42.4% (122/288) for hip pain, and 38.4% (188/490) for knee pain after 4 weeks (Figure 4; Multimedia Appendix 7). After 12 weeks, the retention rates ranged from 8% (acute lower back pain and chronic upper back pain) to 20% (acute knee pain), with an average of 14% (Multimedia Appendix 7).

## Discussion

### Principal Findings

The data showed improvements in the primary outcome, as indicated by a significant decrease in overall pain intensity, and most of the secondary outcomes (pain area; pain area by pain duration, as assessed with a VNRS; and function scores). A reduction in acute pain intensity was only observed in patients with lower back pain, while we observed no significant changes in the remaining patients. Under the assumption that providers did not prescribe Vivira for conditions not covered by the approved spectrum of conditions, we hypothesized that most acute pain episodes in the hip and knee reflected acute exacerbations of pre-existing structural and degenerative conditions (eg, activated osteoarthritis) that cannot be addressed sufficiently with only a self-directed home exercise program. Yet, conclusions based on the limited sample size of the hip and knee groups warrant careful consideration.

A statistically significant change in any patient-reported outcome does not per se reflect a clinically meaningful effect. It, therefore, needs to be discussed whether changes in pain scores also reflect a clinically significant change. For acute pain, related work has established a robust equivalence of different pain scores and its response to different therapeutic interventions. Holdgate et al [20] estimated pain score reductions between 1.2 and 1.6 points on a VNRS to be within a minimum clinically significant difference. For chronic pain, a broad consensus has been established that a clinically important difference can be assumed if pain reduction of >30% from the initial pain intensity is achieved [21,22]. Applying these thresholds to the data at hand, we conclude that the achieved pain score reductions after 2, 4, 8, and 12 weeks (reduction of 1.94 points) are well in the range of the minimum clinically significant difference. After 12 weeks, a >30% reduction in the pain intensity was seen in all pain areas, and most pain areas (upper back, hip, and knee) also crossed the clinically important difference threshold. However, it remains to be discussed why the effect of the app levels off to a steady state after the early use phase. We hypothesize that the mainly unspecific and degenerative musculoskeletal conditions are effectively addressed by a constant exercise intensity that can be maintained over a long period, as shown in the existing literature [23,24]. Additionally, we used interdisciplinary expert consensus from a panel of orthopedic surgeons and physiotherapists, as well as data from a randomized controlled trial of the home exercise program to assess the plausibility of the results from this study [25]. However, owing to the high attrition in the data set, careful interpretation of potential biases is warranted.

### Secondary Results

In line with the improvements in pain intensity, we saw significant improvements in the function scores for most indication subgroups and pain durations. These improvements were particularly emphasized for chronic conditions. Interestingly, the responses for hip conditions in general and acute hip pain in particular were not of a relevant magnitude, except for the subacute and chronic strength scores. We attribute this to the fact that most patients in this category had osteoarthritis or other degenerative conditions of the hip joint, which are typically associated with a much greater limitation in the range of movement compared to, for example, degenerative conditions of the knee. Additionally, an episode of acute pain in any degenerative musculoskeletal condition likely reflects an exacerbation, and an exercise program might not provide the ideal therapeutic intervention for this context. Additionally, we are aware of the small sample size of this subgroup and hence consider the explanatory power of this subgroup analysis as greatly limited. A second noteworthy aspect centers around the assessment of coordination, which only demonstrated significant improvements in patients with chronic lower back pain, chronic upper back pain, and chronic knee pain. In comparison with the strength and mobility assessments, which showed significant improvements across most indication subgroups and pain durations, the limited performance of the coordination dimension reflects either an insufficient stimulus to improve coordination through the individualized exercise program or a much more consolidated deficit in coordination that lags behind the responses in the strength and mobility scores. In line with the principles of regional interdependence, we consider the latter plausible [15,16]. Consequently, we saw more sustained responses in the coordination score for prolonged use phases among patients with chronic pain across all pain areas (Multimedia Appendix 5 and Multimedia Appendix 6). Another aspect addresses the patterns of improvement over the time of use. In contrast to pain score reductions, which leveled off after the early use phase and were primarily maintained during the subsequent maintenance use phase, we saw a continued improvement in the function scores reported (Multimedia Appendix 5 and Multimedia Appendix 6).

### Strengths and Limitations

The strengths of this study are the large real-world prescription data set and the use of the first prescription-based postmarketing data available from a DiGA for musculoskeletal conditions within the regulatory framework of the DVG. The findings provide insights into the clinical effects expected in a real-world care setting and highlight the methodological challenges of complex patient-reported data sets. The importance of these data for the thorough assessment of novel and digital therapeutics has been underscored by the introduction of the United States Twenty-First Century Cures Act in 2016 and the communicated position by the European Medicines Agency [26,27]. Yet, there are some relevant limitations in our study that primarily affect the external validity of our findings. First, the enrollment was assumed to follow a relative self-selection mechanism, which introduced a relevant selection bias that we could not control, given the study design. Second, and although our data showed above-average retention rates, we noted a relevant loss to follow-up across all strata, which is probably of differential nature. Yet, this is not unexpected, as related work has also reported a significant decline in participation in digital health applications [28,29], and we consider it an adequate reflection of the current real-world pattern of use. Additional limitations due to the real-world setting are that we were not able to record any medical history from participants regarding other events that may have affected the initial pain, the development of pain intensity, or the potential effects of the measurements (including but not limited to the occurrence of other physical or psychological diseases) or maturation of the patients (eg, coming to terms with constant or chronic pain and developing coping mechanisms that might influence the perception of pain intensity). Furthermore, we could not measure how familiar the patients were with digital interventions. Since patients can also improve owing to other uncontrollable factors, we need to acknowledge a potential regression toward the mean. As in most observational studies, measuring outcomes may influence the outcomes. However, since the measurements were included in the intervention itself, we would argue that this effect is smaller in this study compared with other work in which measurements were conducted outside of the intervention (eg, pre-post examinations of physicians). Lastly, we consider the consensus-based discrete transformation of the binary results of the movement assessment as methodologically challenging, and thus, a quantitative validation is required.

### Comparison With Prior Work

This work complements preliminary use data of the same digital home exercise program published elsewhere [30]. While the preliminary data also demonstrated a significant and clinically relevant reduction in pain intensity prior to stratification, this study allows a more robust interpretation at the indication-specific level and shows significant improvements in pain intensity for patients with upper and lower back pain, as well as for subpopulations with subacute and chronic hip and knee pain. Although the retention rates in this analysis are considerably higher than the rates in the preliminary analysis, this study suffers from high and probably differential loss to follow-up, which may result in selection bias. Retention, which is required to enable a sufficiently granular analysis of the use and outcome data, is a well-described problem of digital therapeutics. Baumel et al [29] reported an average 30-day retention rate of 3.3% (IQR 6.2%) for all digital health applications examined. Although their analysis was limited to digital therapeutics for mental health conditions and included only those applications that were freely available on the internet and in established online stores (ie, Google Play Store), it exemplifies the stereotypical retention curve of many digital therapeutics well and underscores the common challenge of increasing the retention rate for digital therapeutics. In comparison to these data, our study showed above-average retention rates (Figure 4; Multimedia Appendix 7). We know, however, that free-to-use digital health applications likely have different interaction and retention dynamics than DiGAs and comparable prescription digital health applications. Pratap et al [28] identified (1) required prescription by a physician or psychotherapist, (2) presence of at least one specified condition, and (3) middle to old age as factors that contribute to higher retention rates. From our perspective, all factors were met for our investigation of Vivira. We, therefore, assume that average retention rates are likely to be significantly higher among DiGAs than among free-to-use digital health applications, although further research needs to yield the required evidence. In addition, patient perception of effectiveness and gamification elements can probably contribute to a higher retention rate, although the available evidence in this field needs to be substantiated further [31,32].

### Conclusions

Digital therapeutics can offer accessible and readily available therapeutic means at scale to effectively address the increasing demand for care arising from unspecific and degenerative musculoskeletal conditions. This work presents the first postmarketing data to demonstrate the real-world effects of a digital prescription home exercise program under the DVG for a broad spectrum of unspecific and degenerative musculoskeletal conditions. The demonstration of statistically significant and clinically relevant effects is crucial to establish digital therapeutics as a therapeutic option in the field of musculoskeletal health. As reported in this study, complex user-reported observational data pose analytical challenges and have not yet become a standard feature in the evaluation process of digital therapeutics. Nevertheless, these data will likely complement confirmatory trial data for the clinical and regulatory assessment of the effectiveness of digital therapeutics.

## Appendix

Multimedia Appendix 1 Cross table from the chi-square test for pain duration by age.

Multimedia Appendix 2 Cross table from the chi-square test for pain area by age.

Multimedia Appendix 3 Cross table from the chi-square test for pain duration by sex.

Multimedia Appendix 4 Cross table from the chi-square test for pain area by sex.

Multimedia Appendix 5 Self-reported functional scores and changes across indication subsets and reported pain durations by retained days in matched comparisons between the first and third completed functional assessments.

Multimedia Appendix 6 Self-reported functional scores and changes across indication subsets and reported pain durations by retained days in matched comparisons between the first and fourth completed functional assessments.

Multimedia Appendix 7 User retention across indication subsets and reported pain durations.

## Abbreviations

DiGA: Digitale Gesundheitsanwendung (Digital Health Application)
DVG: Digitale-Versorgung-Gesetz (Digital Health Care Act)
VNRS: verbal numerical rating scale
