# Supplementary material for: Postmarketing Follow-Up of a Digital Home Exercise Program for Back, Hip, and Knee Pain: Retrospective Observational Study With a Time-Series and Matched-Pair Analysis
Source: J Med Internet Res. 2023 Feb 27;25:e43775. doi: 10.2196/43775 (PMC10012010; doi:10.2196/43775)
Supplement: Multimedia Appendix 1 [file jmir_v25i1e43775_app1.docx]

Multimedia Appendix 1. Cross table from the chi-square test for pain duration by age.

| **Age Group** | **Chi-Test Values** | **Acute** | **Subacute** | **Chronic** | **Row Total** |
| --- | --- | --- | --- | --- | --- |
| **18-35** | Count | 124 | 180 | 587 | 891 |
|  | Expected Values | 105.575 | 170.392 | 615.033 |  |
|  | Row Percent | 13.92% | 20.20% | 65.88% | 24.55% |
|  | Std Residual | 1.793 | 0.736 | -1.13 |  |
| **36-45** | Count | 102 | 122 | 448 | 672 |
|  | Expected Values | 79.625 | 128.511 | 463.863 |  |
|  | Row Percent | 15.18% | 18.16% | 66.67% | 18.52% |
|  | Std Residual | 2.507 | -0.574 | -0.737 |  |
| **46-55** | Count | 106 | 187 | 661 | 954 |
|  | Expected Values | 113.039 | 182.44 | 658.52 |  |
|  | Row Percent | 11.11% | 19.60% | 69.29% | 26.29% |
|  | Std Residual | -0.662 | 0.338 | 0.097 |  |
| **56-65** | Count | 73 | 147 | 593 | 813 |
|  | Expected Values | 96.332 | 155.476 | 561.192 |  |
|  | Row Percent | 8.98% | 18.08% | 72.94% | 22.40% |
|  | Std Residual | -2.377 | -0.68 | 1.343 |  |
| **66-75** | Count | 21 | 49 | 178 | 248 |
|  | Expected Values | 29.386 | 47.427 | 171.188 |  |
|  | Row Percent | 8.47% | 19.76% | 71.77% | 6.83% |
|  | Std Residual | -1.547 | 0.228 | 0.521 |  |
| **75+** | Count | 4 | 9 | 38 | 51 |
|  | Expected Values | 6.043 | 9.753 | 35.204 |  |
|  | Row Percent | 7.84% | 17.65% | 74.51% | 1.41% |
|  | Std Residual | -0.831 | -0.241 | 0.471 |  |
| **Column Total** |  | 430 | 694 | 2505 | 3629 |
