# Supplementary material for: Postmarketing Follow-Up of a Digital Home Exercise Program for Back, Hip, and Knee Pain: Retrospective Observational Study With a Time-Series and Matched-Pair Analysis
Source: J Med Internet Res. 2023 Feb 27;25:e43775. doi: 10.2196/43775 (PMC10012010; doi:10.2196/43775)
Supplement: Multimedia Appendix 2 [file jmir_v25i1e43775_app2.docx]

Multimedia Appendix 2. Cross table from the chi-square test for pain area by age.

| **Age Group** | **Chi-Test Values** | **Lower Back** | **Upper Back** | **Hip** | **Knee** | **Row Total** |
| --- | --- | --- | --- | --- | --- | --- |
| **18-35** | Count | 397 | 343 | 46 | 105 | 891 |
|  | Expected Values | 403.147 | 296.836 | 70.71 | 120.306 |  |
|  | Row Percent | 44.56% | 38.50% | 5.16% | 11.79% | 24.55% |
|  | Std Residual | -0.306 | 2.679 | -2.939 | -1.395 |  |
| **36-45** | Count | 313 | 257 | 36 | 66 | 672 |
|  | Expected Values | 304.057 | 223.877 | 53.33 | 90.736 |  |
|  | Row Percent | 46.58% | 38.24% | 5.36% | 9.82% | 18.52% |
|  | Std Residual | 0.513 | 2.214 | -2.373 | -2.597 |  |
| **46-55** | Count | 425 | 302 | 97 | 130 | 954 |
|  | Expected Values | 431.653 | 317.825 | 75.71 | 128.812 |  |
|  | Row Percent | 44.55% | 31.66% | 10.17% | 13.63% | 26.29% |
|  | Std Residual | -0.32 | -0.888 | 2.447 | 0.105 |  |
| **56-65** | Count | 386 | 229 | 75 | 123 | 813 |
|  | Expected Values | 367.855 | 270.851 | 64.52 | 109.774 |  |
|  | Row Percent | 47.48% | 28.17% | 9.23% | 15.13% | 22.40% |
|  | Std Residual | 0.946 | -2.543 | 1.305 | 1.262 |  |
| **66-75** | Count | 101 | 65 | 28 | 54 | 248 |
|  | Expected Values | 112.212 | 82.621 | 19.681 | 33.486 |  |
|  | Row Percent | 40.73% | 26.21% | 11.29% | 21.77% | 6.83% |
|  | Std Residual | -1.058 | -1.939 | 1.875 | 3.545 |  |
| **75+** | Count | 20 | 13 | 6 | 12 | 51 |
|  | Expected Values | 23.076 | 16.991 | 4.047 | 6.886 |  |
|  | Row Percent | 39.22% | 25.49% | 11.77% | 23.53% | 1.41% |
|  | Std Residual | -0.64 | -0.968 | 0.971 | 1.949 |  |
| **Column Total** |  | 1642 | 1209 | 288 | 490 | 3629 |
