# Supplementary material for: Postmarketing Follow-Up of a Digital Home Exercise Program for Back, Hip, and Knee Pain: Retrospective Observational Study With a Time-Series and Matched-Pair Analysis
Source: J Med Internet Res. 2023 Feb 27;25:e43775. doi: 10.2196/43775 (PMC10012010; doi:10.2196/43775)
Supplement: Multimedia Appendix 3 [file jmir_v25i1e43775_app3.docx]

Multimedia Appendix 3. Cross table from the chi-square test for pain duration by sex.

| **Sex** | **Chi-Test Values** | **Acute** | **Subacute** | **Chronic** | **Row Total** |
| --- | --- | --- | --- | --- | --- |
| **Female** | Count | 281 | 490 | 1836 | 2607 |
|  | Expected Values | 308.903 | 498.556 | 1799.541 |  |
|  | Row Percent | 10.78% | 18.80% | 70.43% | 71.84% |
|  | Std Residual | -1.588 | -0.383 | 0.859 |  |
| **Male** | Count | 149 | 204 | 669 | 1022 |
|  | Expected Values | 121.097 | 195.444 | 705.459 |  |
|  | Row Percent | 14.58% | 19.96% | 65.46% | 28.16% |
|  | Std Residual | 2.536 | 0.612 | -1.373 |  |
| **Column Total** |  | 430 | 694 | 2505 | 3629 |
