# Supplementary material for: Postmarketing Follow-Up of a Digital Home Exercise Program for Back, Hip, and Knee Pain: Retrospective Observational Study With a Time-Series and Matched-Pair Analysis
Source: J Med Internet Res. 2023 Feb 27;25:e43775. doi: 10.2196/43775 (PMC10012010; doi:10.2196/43775)
Supplement: Multimedia Appendix 4 [file jmir_v25i1e43775_app4.docx]

Multimedia Appendix 4. Cross table from the chi-square test for pain area by sex.

| **Sex** | **Chi-Test Values** | **Lower Back** | **Upper Back** | **Hip** | **Knee** | **Row Total** |
| --- | --- | --- | --- | --- | --- | --- |
| **Female** | Count | 1113 | 931 | 221 | 342 | 2607 |
|  | Expected Values | 1179.579 | 868.521 | 206.893 | 352.006 |  |
|  | Row Percent | 42.69% | 35.71% | 8.48% | 13.12% | 71.84% |
|  | Std Residual | -1.939 | 2.12 | 0.981 | -0.533 |  |
| **Male** | Count | 529 | 278 | 67 | 148 | 1022 |
|  | Expected Values | 462.421 | 340.479 | 81.107 | 137.994 |  |
|  | Row Percent | 51.76% | 27.20% | 6.56% | 14.48% | 28.16% |
|  | Std Residual | 3.096 | -3.386 | -1.566 | 0.852 |  |
| **Column Total** |  | 1642 | 1209 | 288 | 490 | 3629 |
