# Supplementary material for: Postmarketing Follow-Up of a Digital Home Exercise Program for Back, Hip, and Knee Pain: Retrospective Observational Study With a Time-Series and Matched-Pair Analysis
Source: J Med Internet Res. 2023 Feb 27;25:e43775. doi: 10.2196/43775 (PMC10012010; doi:10.2196/43775)
Supplement: Multimedia Appendix 5 [file jmir_v25i1e43775_app5.docx]

**Multimedia Appendix 5.** Self-reported functional scores and changes across indication subsets and reported pain durations by retained days in matched comparisons between the first and third completed functional assessments.

| **Reported Pain Area** | | **Lower Back** | | | **Upper Back** | | | **Hip** | | | **Knee** | | |
| --- | --- | --- | --- | --- | --- | --- | --- | --- | --- | --- | --- | --- | --- |
|  |  | **Acute** | **Subacute** | **Chronic** | **Acute** | **Subacute** | **Chronic** | **Acute** | **Subacute** | **Chronic** | **Acute** | **Subacute** | **Chronic** |
| **Total Score** | **N** | 16 | 28 | 146 | 12 | 18 | 100 | 4 | 9 | 35 | 4 | 19 | 53 |
|  | **Retained Days (IQR)** | 58,5 (47; 61,5) | 49 (31; 61,5) | 58 (44; 63) | 58 (52; 63,5) | 57 (38; 58) | 56 (30,5; 60) | 32,5 (20; 47) | 57 (30; 63) | 58 (47; 62) | 57,5 (56,5; 58,5) | 57 (43; 62) | 57 (47; 62) |
|  | **Initial (IQR)** | 67 (50; 77) | 63 (47; 73) | 57 (40; 73) | 67 (53; 73) | 60 (37; 77) | 53 (33; 67) | 65 (48,5; 70) | 55 (43; 67) | 63 (50; 73) | 53 (45; 65) | 63 (55; 73) | 60 (47; 70) |
|  | **Last (IQR)** | 87 (78,5; 97) | 70 (53,5; 87) | 67 (53; 80) | 67 (55; 78,5) | 67 (47; 83) | 63 (47; 77) | 73,5 (50; 90) | 73 (63; 83) | 73 (63; 83) | 83,5 (80; 93,5) | 73 (67; 87) | 73 (57; 80) |
|  | **Test** | ** | * | *** | ns | ** | *** | ns | ** | ** | ns | ** | *** |
| **Strength Score** | **N** | 16 | 28 | 146 | 12 | 18 | 100 | 4 | 9 | 35 | 4 | 19 | 53 |
|  | **Retained Days (IQR)** | 58,5 (47; 61,5) | 49 (31; 61,5) | 58 (44; 63) | 58 (52; 63,5) | 57 (38; 58) | 56 (30,5; 60) | 32,5 (20; 47) | 57 (30; 63) | 58 (47; 62) | 57,5 (56,5; 58,5) | 57 (43; 62) | 57 (47; 62) |
|  | **Last (IQR)** | 95 (80; 100) | 80 (35; 100) | 70 (40; 90) | 70 (55; 80) | 75 (50; 100) | 60 (40; 85) | 60 (30; 90) | 70 (60; 100) | 80 (60; 100) | 100 (80; 100) | 90 (70; 100) | 70 (50; 90) |
|  | **Test** | * | ns | *** | ns | ns | * | ns | ns | * | ns | * | ** |
| **Mobility Score** | **N** | 16 | 28 | 146 | 12 | 18 | 100 | 4 | 9 | 35 | 4 | 19 | 53 |
|  | **Retained Days (IQR)** | 58,5 (47; 61,5) | 49 (31; 61,5) | 58 (44; 63) | 58 (52; 63,5) | 57 (38; 58) | 56 (30,5; 60) | 32,5 (20; 47) | 57 (30; 63) | 58 (47; 62) | 57,5 (56,5; 58,5) | 57 (43; 62) | 57 (47; 62) |
|  | **Initial (IQR)** | 70 (55; 80) | 67,5 (45; 80) | 60 (45; 75) | 65 (55; 75) | 60 (40; 75) | 50 (35; 70) | 67,5 (52,5; 85) | 57,5 (50; 70) | 60 (50; 70) | 60 (52,5; 70) | 60 (50; 70) | 60 (45; 70) |
|  | **Last (IQR)** | 87,5 (80; 90) | 65 (55; 90) | 65 (50; 80) | 67,5 (57,5; 75) | 70 (50; 80) | 60 (40; 75) | 80 (50; 100) | 65 (60; 90) | 70 (55; 80) | 85 (75; 95) | 70 (60; 80) | 70 (55; 80) |
|  | **Test** | * | ns | *** | ns | * | *** | ns | ns | * | ns | * | *** |
| **Coordination Score** | **N** | 16 | 28 | 146 | 12 | 18 | 100 | 4 | 9 | 35 | 4 | 19 | 53 |
|  | **Retained Days (IQR)** | 58,5 (47; 61,5) | 49 (31; 61,5) | 58 (44; 63) | 58 (52; 63,5) | 57 (38; 58) | 56 (30,5; 60) | 32,5 (20; 47) | 57 (30; 63) | 58 (47; 62) | 57,5 (56,5; 58,5) | 57 (43; 62) | 57 (47; 62) |
|  | **Initial (IQR)** | 70 (60; 80) | 70 (50; 80) | 65 (40; 80) | 80 (60; 80) | 60 (40; 80) | 60 (40; 80) | 80 (60; 80) | 60 (30; 80) | 60 (40; 80) | 55 (35; 60) | 60 (60; 80) | 60 (40; 80) |
|  | **Last (IQR)** | 80 (80; 100) | 75 (40; 100) | 80 (40; 80) | 80 (60; 85) | 65 (40; 80) | 70 (45; 80) | 80 (40; 100) | 80 (60; 80) | 80 (60; 80) | 80 (70; 90) | 80 (60; 80) | 60 (40; 80) |
|  | **Test** | ns | ns | *** | ns | ns | ** | ns | ns | * | ns | * | ** |

Adjusted for familywise error, p < 0.0167 = *, p < 0.00167 = **, p < 0.000167 = ***.
