# Supplementary material for: Postmarketing Follow-Up of a Digital Home Exercise Program for Back, Hip, and Knee Pain: Retrospective Observational Study With a Time-Series and Matched-Pair Analysis
Source: J Med Internet Res. 2023 Feb 27;25:e43775. doi: 10.2196/43775 (PMC10012010; doi:10.2196/43775)
Supplement: Multimedia Appendix 6 [file jmir_v25i1e43775_app6.docx]

Multimedia Appendix 6. Self-reported functional scores and changes across indication subsets and reported pain durations by retained days in matched comparisons between the first and fourth completed functional assessments.

| **Reported Pain Area** | | **Lower Back** | | | **Upper Back** | | | **Hip** | | | **Knee** | | |
| --- | --- | --- | --- | --- | --- | --- | --- | --- | --- | --- | --- | --- | --- |
|  |  | **Acute** | **Subacute** | **Chronic** | **Acute** | **Subacute** | **Chronic** | **Acute** | **Subacute** | **Chronic** | **Acute** | **Subacute** | **Chronic** |
| **Total Score** | **N** | 5 | 15 | 55 | 2 | 5 | 50 | 1 | 3 | 19 | 3 | 9 | 27 |
|  | **Retained Days (IQR)** | 85 (63; 139) | 61 (42; 68) | 65 (45; 86) | 87 (86; 88) | 80 (63; 81) | 68 (38; 85) | 49 (49; 49) | 75 (60; 85) | 84 (64; 87) | 85 (81; 94) | 81 (72; 90) | 85 (70; 90) |
|  | **Initial (IQR)** | 67 (50; 77) | 63 (47; 73) | 57 (40; 73) | 67 (53; 73) | 60 (37; 77) | 53 (33; 67) | 65 (48,5; 70) | 55 (43; 67) | 63 (50; 73) | 53 (45; 65) | 63 (55; 73) | 60 (47; 70) |
|  | **Last (IQR)** | 77 (73; 90) | 80 (60; 93) | 73 (53; 83) | 85 (70; 100) | 67 (43; 70) | 65 (43; 80) | 73 (73; 73) | 87 (70; 100) | 80 (63; 87) | 87 (73; 93) | 80 (80; 83) | 67 (50; 83) |
|  | **Test** | ns | * | *** | ns | ns | *** | ns | ns | * | ns | ns | ** |
| **Strength Score** | **N** | 5 | 15 | 55 | 2 | 5 | 50 | 1 | 3 | 19 | 3 | 9 | 27 |
|  | **Retained Days (IQR)** | 85 (63; 139) | 61 (42; 68) | 65 (45; 86) | 87 (86; 88) | 80 (63; 81) | 68 (38; 85) | 49 (49; 49) | 75 (60; 85) | 84 (64; 87) | 85 (81; 94) | 81 (72; 90) | 85 (70; 90) |
|  | **Initial (IQR)** | 60 (40; 80) | 50 (30; 70) | 60 (30; 70) | 60 (50; 80) | 50 (20; 80) | 50 (20; 80) | 45 (40; 70) | 55 (20; 60) | 60 (40; 80) | 20 (45; 70) | 80 (60; 80) | 60 (40; 80) |
|  | **Last (IQR)** | 80 (60; 90) | 60 (50; 100) | 70 (50; 100) | 95 (90; 100) | 70 (60; 80) | 60 (40; 80) | 40 (40; 40) | 100 (60; 100) | 80 (70; 100) | 100 (80; 100) | 90 (80; 100) | 60 (50; 100) |
|  | **Test** | ns | * | * | ns | ns | * | ns | ns | ns | ns | ns | ns |
| **Mobility Score** | **N** | 5 | 15 | 55 | 2 | 5 | 50 | 1 | 3 | 19 | 3 | 9 | 27 |
|  | **Retained Days (IQR)** | 85 (63; 139) | 61 (42; 68) | 65 (45; 86) | 87 (86; 88) | 80 (63; 81) | 68 (38; 85) | 49 (49; 49) | 75 (60; 85) | 84 (64; 87) | 85 (81; 94) | 81 (72; 90) | 85 (70; 90) |
|  | **Initial (IQR)** | 70 (55; 80) | 67,5 (45; 80) | 60 (45; 75) | 65 (55; 75) | 60 (40; 75) | 50 (35; 70) | 67,5 (52,5; 85) | 57,5 (50; 70) | 60 (50; 70) | 60 (52,5; 70) | 60 (50; 70) | 60 (45; 70) |
|  | **Last (IQR)** | 90 (85; 90) | 80 (60; 90) | 70 (55; 90) | 80 (60; 100) | 55 (50; 65) | 62,5 (45; 80) | 90 (90; 90) | 100 (55; 100) | 80 (60; 85) | 80 (70; 90) | 75 (70; 80) | 70 (50; 80) |
|  | **Test** | ns | ns | *** | ns | ns | *** | ns | ns | ns | ns | * | ** |
| **Coordination Score** | **N** | 5 | 15 | 55 | 2 | 5 | 50 | 1 | 3 | 19 | 3 | 9 | 27 |
|  | **Retained Days (IQR)** | 85 (63; 139) | 61 (42; 68) | 65 (45; 86) | 87 (86; 88) | 80 (63; 81) | 68 (38; 85) | 49 (49; 49) | 75 (60; 85) | 84 (64; 87) | 85 (81; 94) | 81 (72; 90) | 85 (70; 90) |
|  | **Initial (IQR)** | 70 (60; 80) | 70 (50; 80) | 65 (40; 80) | 80 (60; 80) | 60 (40; 80) | 60 (40; 80) | 60 (55; 80) | 60 (30; 80) | 60 (40; 80) | 55 (35; 60) | 60 (50; 80) | 60 (40; 80) |
|  | **Last (IQR)** | 70 (70; 100) | 90 (40; 100) | 80 (50; 90) | 90 (80; 100) | 60 (40; 60) | 60 (40; 80) | 80 (80; 80) | 100 (50; 100) | 70 (60; 90) | 80 (60; 80) | 80 (80; 90) | 60 (40; 80) |
|  | **Test** | ns | ns | *** | ns | ns | *** | ns | ns | ns | ns | * | ** |

Adjusted for familywise error, p < 0.0167 = *, p < 0.00167 = **, p < 0.000167 = ***.
