# Supplementary material for: Postmarketing Follow-Up of a Digital Home Exercise Program for Back, Hip, and Knee Pain: Retrospective Observational Study With a Time-Series and Matched-Pair Analysis
Source: J Med Internet Res. 2023 Feb 27;25:e43775. doi: 10.2196/43775 (PMC10012010; doi:10.2196/43775)
Supplement: Multimedia Appendix 7 [file jmir_v25i1e43775_app7.docx]

Multimedia Appendix 7. User retention across indication subsets and reported pain durations.

| **Indication**  **Subset** | **Pain Duration** | **Initial**  **(N, %)** | **Week 2**  **(N, %)** | **Week 4**  **(N, %)** | **Week 8**  **(N, %)** | **Week 12**  **(N, %)** |
| --- | --- | --- | --- | --- | --- | --- |
| **All** | All | 3629 (100%) | 1776 (49%) | 1330 (37%) | 820 (23%) | 458 (13%) |
| **Lower Back** | All | 1642 (100%) | 812 (49%) | 601 (37%) | 376 (23%) | 207 (13%) |
|  | Acute | 231 (100%) | 117 (51%) | 66 (29%) | 42 (18%) | 19 (8%) |
|  | Subacute | 314 (100%) | 149 (47%) | 112 (36%) | 64 (20%) | 31 (10%) |
|  | Chronic | 1097 (100%) | 546 (50%) | 423 (39%) | 270 (25%) | 157 (14%) |
| **Upper Back** | All | 1209 (100%) | 573 (47%) | 419 (35%) | 249 (21%) | 129 (11%) |
|  | Acute | 140 (100%) | 61 (44%) | 52 (37%) | 28 (20%) | 18 (13%) |
|  | Subacute | 207 (100%) | 94 (45%) | 75 (36%) | 35 (17%) | 22 (11%) |
|  | Chronic | 862 (100%) | 418 (48%) | 292 (34%) | 186 (22%) | 89 (10%) |
| **Hip** | All | 288 (100%) | 159 (55%) | 122 (42%) | 73 (25%) | 45 (16%) |
|  | Acute | 24 (100%) | 11 (46%) | 14 (58%) | 7 (29%) | 4 (17%) |
|  | Subacute | 66 (100%) | 38 (58%) | 26 (39%) | 12 (18%) | 10 (15%) |
|  | Chronic | 198 (100%) | 110 (56%) | 82 (41%) | 54 (27%) | 31 (16%) |
| **Knee** | All | 490 (100%) | 232 (47%) | 188 (38%) | 122 (25%) | 77 (16%) |
|  | Acute | 35 (100%) | 15 (43%) | 14 (40%) | 9 (26%) | 7 (20%) |
|  | Subacute | 107 (100%) | 52 (49%) | 36 (34%) | 23 (21%) | 13 (12%) |
|  | Chronic | 348 (100%) | 165 (47%) | 138 (40%) | 90 (26%) | 57 (16%) |
